# Optimization Design of Casting Process for Large Long Lead Cylinder of Aluminum Alloy

**DOI:** 10.3390/ma18030531

**Published:** 2025-01-24

**Authors:** Liang Huang, Yan Cao, Mengfei Zhang, Zhichao Meng, Tuo Wang, Xiaozhe Zhu

**Affiliations:** 1 School of Mechatronic Engineering, Xi’an Technological University, No. 2 Xuefu Middle Street, Weiyang District, Xi’an 710021, China; zhangmengfei202202@163.com (M.Z.); mengzhichao2024@163.com (Z.M.); 2 School of Computer Science and Engineering, Xi’an Technological University, No. 2 Xuefu Middle Street, Weiyang District, Xi’an 710021, China; caoyan@xatu.edu.cn; 3 Advanced Manufacturing Research Institute, Xi’an KunLun Industry (Group) Company with Limited Liability, No. 67 Xingfu North Street, Xincheng District, Xi’an 710043, China; wangyumfei@139.com (T.W.); zhu_xz2003@163.com (X.Z.)

**Keywords:** aluminum alloy large long lead cylinder, low pressure casting, numerical simulation, response surface method

## Abstract

As the core component of chain-less ammunition transmission system, the large long lead cylinder adopts ZL205A alloy, which has the advantages of high strength and wear resistance. However, in its main casting production process, the forming quality is mainly determined by the casting process parameters under the premise of determining a reasonable casting system. Considering that the casting process parameters are the process feedback expression of the macroscopic forming quality and comprehensive mechanical properties by controlling the coupling effect of the metal liquid flow in the microscopic flow field, the directional solidification crystallization of the alloy and the solid–liquid heat transfer and heat transfer during the filling and solidification process, the accurate and reasonable selection of casting process parameters is conducive to the stable guarantee of pouring quality. On the basis of the optimized column gap casting system, this study combined numerical simulation and data statistics. Within the rationality of each casting process parameter constructed by single-factor analysis, the response surface method was used to construct a quantitative guidance relationship of each process parameter coupling mapping casting defect, and based on this model, the optimal process parameter combination was realized as follows: compared with traditional metal mold casting and unoptimized low pressure casting, the tensile strength of non-porous casting with holding pressure 14.68 kPa, casting temperature 717.152 °C and mold preheating temperature 256.12 °C increased by 6.6% and 4.1%, respectively, hardness increased by 14.3% and 8.4% respectively, and the elongation is increased by 16.9% and 10.6%, respectively, thus efficiently and accurately improving the process quality.

## 1. Introduction

**Figure 1:**
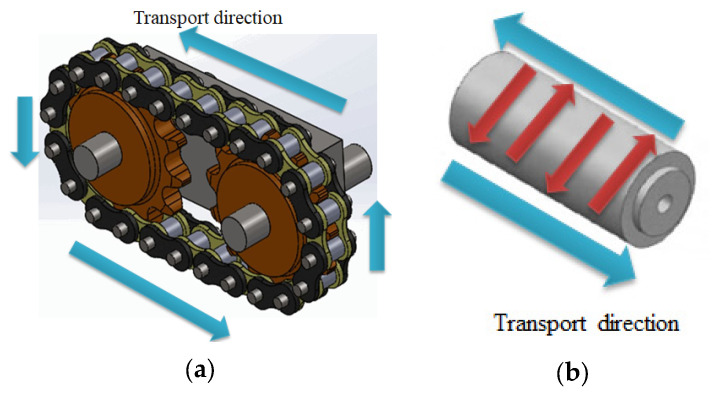
Comparison of two transmission structures: (**a**) sprocket drive system; (**b**) chainless drive system.

**Figure 2:**
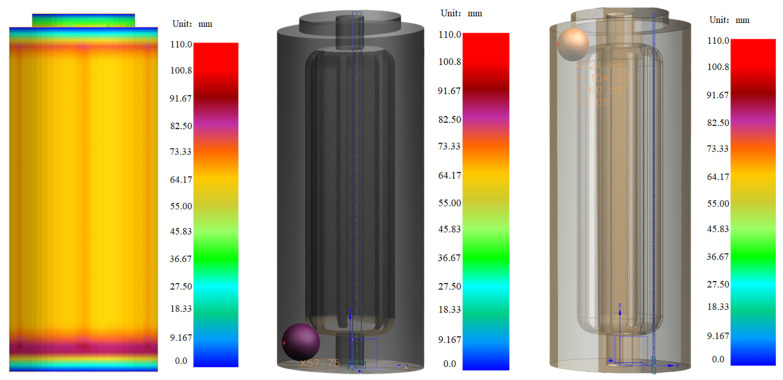
Hot joints distribution map of large long lead cylinder of aluminum alloy.

In recent years, with the rapid development of high-performance equipment materials, ZL205A alloy, as a lightweight alloy independently developed in China, has low mass density, high tensile strength, compressive strength and hardness, good impact resistance and corrosion resistance, and can maintain certain service stability under high temperature environment. At present, it is mainly used in a chain-less ammunition transmission feeding system [1,2,3] (alternate sprocket, as shown in Figure 1). The traditional transmission load is mainly conducted through sprocket, which has the problems of low transmission efficiency and large wear due to the influence of sprocket structure. The new chainless transmission is driven through the grooves on the surface of the cylinder, so compared with the traditional chain transmission has higher transmission load and higher transmission efficiency characteristics. Taking the above-mentioned structure as an example, the characteristics of this large long lead column are as follows: (1) long lead and large cross section of rotary parts, due to the size of processing equipment, currently mainly casting process molding; (2) uneven distribution of wall thickness from top to bottom inside the cylinder (it can be seen that the maximum thickness is at the top and bottom, and the thickness at the bottom is greater than that at the top (as shown in Figure 2)), which is easy to form hot joints, resulting in shrinkage and porosity problems, thermal stress and hot cracking phenomenon. Meanwhile, top-down sequential solidification using low pressure casting can better reduce top casting defects by feeding; (3) a number of reinforcing bars are evenly distributed inside the column, so that if the casting is placed horizontally, the bottom of the casting will have a large plane, which results in the formation of multiple flow paths during the liquid metal pouring process and will involve a large amount of gas when they converge, and is not conducive to the guarantee of molding quality. Therefore, the current pouring system design for the above structure is mainly a column gap pouring system, which is a pouring system that adopts multiple connected gates to achieve continuous flow of metal liquid in the casting process, and is suitable for castings with a large casting height to avoid the problem of too long filling time and unstable filling, so that it has a good temperature gradient to control the solidification rate and reduce the casting defects and improve the casting quality.

After determining an accurate and reasonable pouring system, the casting forming quality is mainly determined by the casting process parameters, for the following reasons: the casting process parameters are the process feedback expression of the macroscopic forming quality and comprehensive mechanical properties by controlling the coupling effect of the metal liquid flow in the microscopic flow field, the directional solidification crystallization of the alloy and the solid–liquid heat transfer and heat transfer during the filling and solidification process. In order to explore the mechanism of action of process parameters on macro-forming quality, researchers explored the mapping relationship between casting process and forming quality under the guidance of process parameters from the perspective of qualitative and quantitative as follows:

In terms of qualitative research, Almonti D et al. (2022) [4] established a finite element analysis model for the filling and solidification process of an aluminum alloy radiator in investment casting, qualitatively established a reasonable range of process parameters and obtained the optimal forming parameters in combination with Niyama criterion, the same method is also reflected in Xu H’s (2019) [5] casting research on aluminum alloy box covers. Meanwhile, Amit C (2022) [6] combined with Design Optimization for Reliability (RBDO) provides guidance for casting process parameters mapping casting defects by establishing a Markov chain probability distribution model for casting failures. Liu S et al. (2021) [7] further proposed a data-driven method for selecting the aluminum alloy component ratio and casting parameters by studying the flow law of metal liquid during mold filling and solidification in aluminum alloy casting. Based on this, Wang JC et al. (2024) [8] established the finite element analysis model of filling and solidification of aluminum alloy DC casting, and established the mechanism of mapping the micro-metal liquid flow field and solidification heat and heat transfer by casting process parameters. Meanwhile, Puga H et al. (2016) [9] used numerical simulation software to simulate the casting process in low pressure sand casting and evaluated its influence on the casting quality by adjusting different process parameters. Zhang ZH et al. (2023) [10] qualitatively realized the guiding relationship of casting process parameters mapping macroscopic comprehensive mechanical properties by deeply studying the microscopic properties of formed structures under different combinations of casting process parameters and establishing two kinds of analytical models: the analytical model between casting parameters and residual stress and the analytical model between shrinkage defects and casting parameters. In terms of the quantitative establishment of research: Ashok RR (2024) [11] and Muthu KR (2023) [12] realized the optimal casting process parameters selection of Al matrix composites with high wear resistance through the Taguchi experiment. Abayomi AA et al. (2022) [13], combined with mechanical experimental data, established the guiding relationship of casting Al6061/glass composite process parameters mapping macroscopic comprehensive mechanical properties by using a statistical method, and obtained the optimal process parameters. He Y et al. (2023) [14] took the low-pressure casting of an aluminum alloy shell casting as the research object and sought to optimize the process parameters to make the casting quality reach the best through modern design methods such as neural network and genetic algorithm on the basis of orthogonal test analysis. Ahmed K (2023) [15] studied the microscopic mechanism and prediction criteria of hot crack formation of ZL205A casting in low pressure casting. By applying artificial intelligence and optimization algorithm, he sought the best combination of process parameters in the search space by simulating biological evolution. Venkata RR (2014) [16] and Nandagopal M (2020) [17] used genetic algorithm and simulated annealing algorithm to explore the global optimal solution of casting quality under the coupling of multiple process parameters. By further optimizing the relaxation factor in the genetic algorithm, Li CX (2024) [18] improved the effectiveness and performance of the prediction model by improving the robustness of the algorithm model. In order to realize intelligent optimization design of process parameters under various requirements, Deng JX et al. (2024) [19] proposed a new intelligent optimization design framework for extrusion casting process parameters based on process data and integrated two-level intelligent integration optimization, and intelligently established imperfectly determined correlations between process parameters and extruded casting quality or characteristics. In order to shorten the prediction model range and improve the prediction accuracy, Natrayan L (2021) [20] used the Taguchi experiment method to analyze the qualitative action mechanism of process parameters, and combined with artificial neural network algorithm to conduct fusion training on experimental mechanical properties, thus obtaining a molding quality prediction model with an accuracy of 95%.

Based on the above literature review, in order to further improve the quality of the pouring process, scholars, respectively, adopted computer numerical simulation analysis, the orthogonal test, and an artificial intelligence algorithm to establish the relationship between pouring process parameters and pouring process quality. However, although the method based on computer numerical simulation can improve the efficiency of optimization design, it does not further analyze the effect of the coupling of various process parameters on the pouring quality by mathematical statistics. On the other hand, based on the analysis of orthogonal experiments and artificial intelligence algorithms, so as to train the intelligent model, a large number of experimental results are introduced, which undoubtedly increases the cost of the research and development cycle of such products. In order to solve this problem, on the basis of the above traditional design of column gap pouring system, the team further optimized the above traditional pouring structure by using ProCast numerical simulation analysis and combined pouring test comparison, including the following: (1) the problem of shrinkage and porosity at the thickest end of the casting has been solved by increasing the reasonable riser height; (2) by increasing the reasonable trapezoid channel width, the crystal grain refinement during filling and solidification is ensured; (3) by increasing the distribution of cold iron in the thickest area to be solidified, the pouring quality of the top of the casting under sequential solidification is improved. On the premise of ensuring the optimal design of the pouring system for the large long lead cylinder of this aluminum alloy, the effect of coupling effects of different process parameters on forming defects was further simulated and analyzed by numerical simulation combined with ProCast 2018, and the mapping relationship of each coupling parameter on forming defects was analyzed by response surface mathematical analysis. Through a series of computer analysis methods, the optimization of the casting process parameters for large long lead cylinders of aluminum alloy is improved in an efficient and accurate way, and finally, it ensures its stable production, and lays a theoretical foundation and technical support for other similar production practices.

## 2. Selection of Process Environment

### 2.1. Pressure Difference Parameter Selection

**Figure 3:**
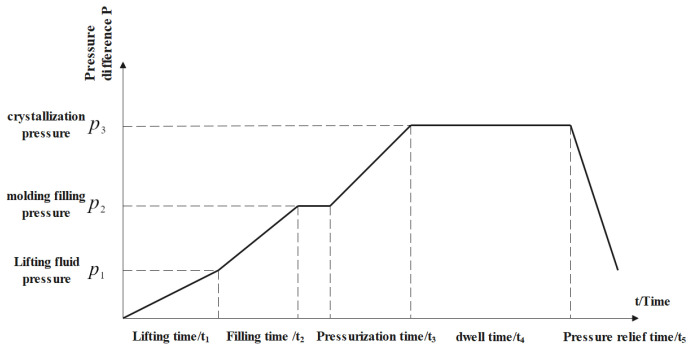
Pressure distribution at each stage of low-pressure casting.

**Figure 4:**
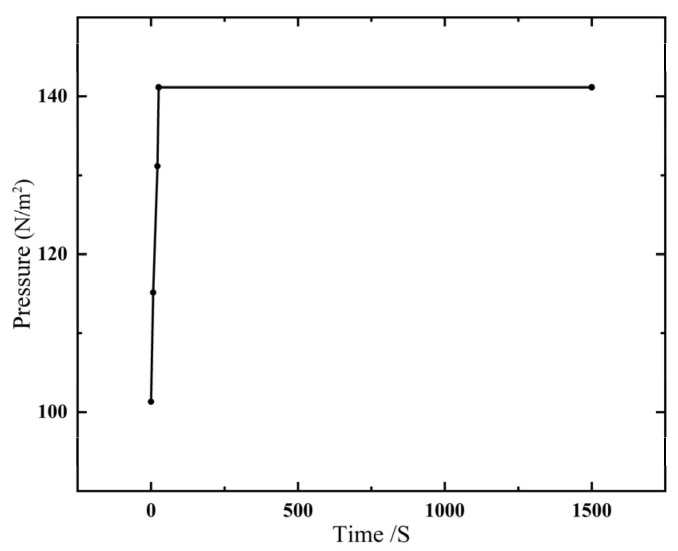
Relationship between pressure and time of low-pressure casting.

In low-pressure casting (anti-gravity casting), the pressure difference parameter is an important parameter to control the metal liquid to overcome gravity from the bottom to top for filling and feeding. If the pressure difference building speed is too large, the filling process will not be stable, and the turbulent flow phenomenon will occur. If the pressure difference building speed is too small, problems such as insufficient pouring, cold isolation, and reduced liquid metal flow will occur, so the pressure and pressure speed of each stage are calculated by PASCAL’s principle, as shown in Figure 3 and Table 1. The obtained pressure difference data are added to the pressure boundary conditions of ProCast database, the corresponding time and pressure value are input, and the pressure difference curve is generated, as shown in Figure 4.

### 2.2. Selection of Initial Pouring Temperature and Preheating Temperature

On the one hand, according to the solid solution characteristics of ZL205A alloy material and the actual production situation, the casting temperature is set to 700 °C. On the other hand, the preheating temperature of the shell with sand casting is 230 °C [21].

### 2.3. Establishment of ZL205A Alloy Material Library

**Figure 5:**
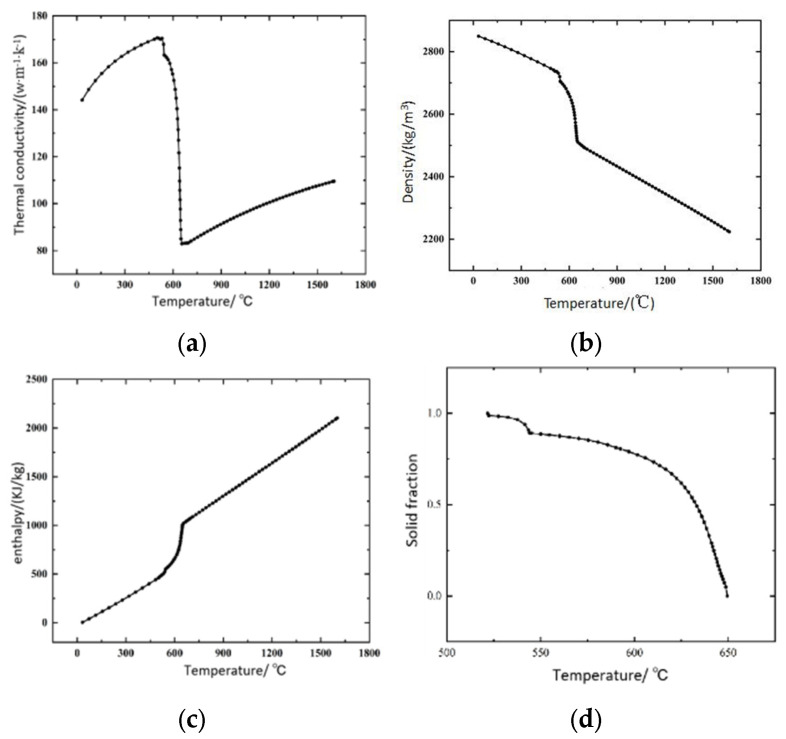
ZL205A material thermal property parameters: (**a**) thermal conductivity; (**b**) density; (**c**) enthalpy of heat; (**d**) solid phase ratio.

The temperature, flow and stress parameters of ZL205A alloy can be obtained by setting the main alloying elements and content of ZL205A alloy in ProCast software 2018 and calculating the thermal physical property parameters according to the software’s own database, as shown in Figure 5.

### 2.4. Setting Boundary Conditions

According to the characteristics of ZL205A alloy, the Scheil model is used to analyze the thermal diffusion model. Considering that the casting will undergo a yield process and become nonlinear when it exceeds the yield limit, the linear elasticity is mainly characterized by Young’s modulus, so there is no yield stress parameter. Therefore, before the stress field analysis, the stress calculation model in this paper should be changed to Elasto-Plastic; Finally, the boundary conditions of the casting are set through the HTC manager of the interface of the ProCast pretreatment module, and the heat transfer coefficient and category of the interface are mainly edited. In the casting process, there is a shrinkage gap between the liquid metal and the mold during the solidification process, and the interfacial heat transfer coefficient is a variable with temperature. The mold studied in this paper is sand mold, so the interfacial heat transfer coefficient decreases with the decrease in temperature during solidification. The ZL205A heat transfer coefficient was added into the database through calculation, and the other heat transfer coefficients of different interfaces are shown in Table 2.

In the further simulation model grid division, the quality of the grid division directly affects the accuracy and stability of the simulation, the more accurate the grid division, the closer it is to the actual production requirements. For complex areas or important stress concentration areas, the density of the mesh needs to be increased to obtain more accurate results. For the common region or the region where the boundary conditions change slowly, the grid density can be appropriately reduced to save computing resources. Therefore, through a large number of comparison experiments in the early stage, the mesh size of the casting system was 0.6 mm, and the mesh size of the other non-casting systems (sand core, riser, etc.) was 1 mm, and the mesh refinement of body mesh and surface mesh was set to high, medium and low, and orthogonal experimental combinations were formed, thus the mesh quality results under different mesh division methods were obtained, as shown in Table 3 (only three iconic groups were listed). The minimum Jacques ratio reflects the degree to which the mesh deviates from the ideal shape and ranges from 0 to 1. The higher the value is, the better the mesh partition quality is. The minimum Jacques ratio ranges from 0.6 to 0.7, which belongs to the acceptable mesh quality. The minimum echo ratio in the range of 0.7–0.8 belongs to good mesh quality. When the minimum Jacobian ratio is greater than 0.9, the mesh quality is very high. Therefore, in combination with Table 3, it can be seen that Plan 1 has the highest grid division solution rate but the worst grid quality; Plan 2 has the higher solution rate and grid quality; and Plan 3 has the highest grid quality (even though the grid refinement greatly increases the solution time but does not significantly improve the grid quality) and the best time, so Plan 3 is selected for grid division.

## 3. Analysis of Numerical Simulation Results of Casting Process

### 3.1. Filling and Solidification Numerical Simulation Analysis of Column Slot Pouring Optimization System

**Figure 6:**
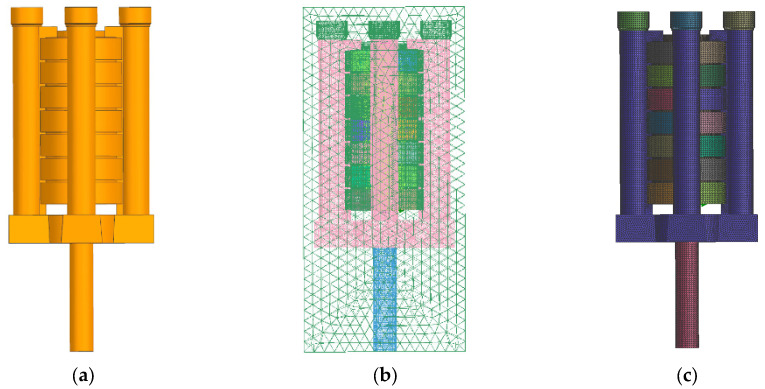
Column slot pouring optimization system and grid division: (**a**) gating system; (**b**) face grid; (**c**) volume grid.

**Figure 7:**
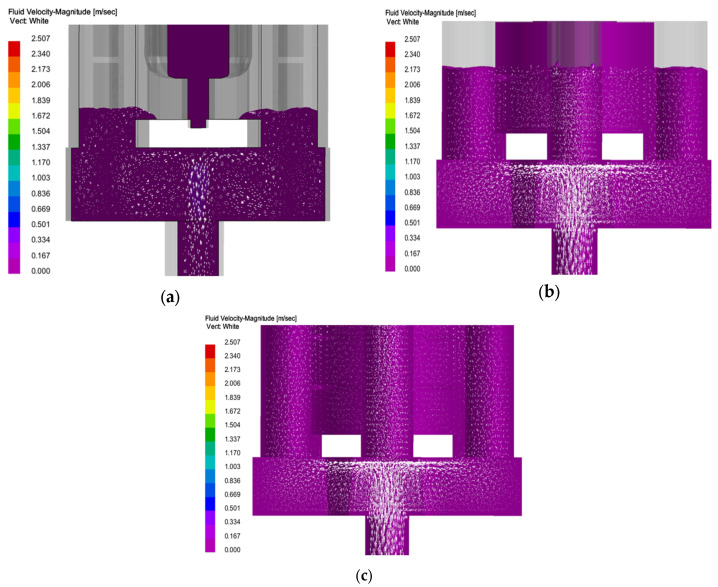
Velocity vector distribution of molten metal during casting: (**a**) *T* = 5 s; (**b**) *T* = 15 s; (**c**) *T* = 30 s.

**Figure 8:**
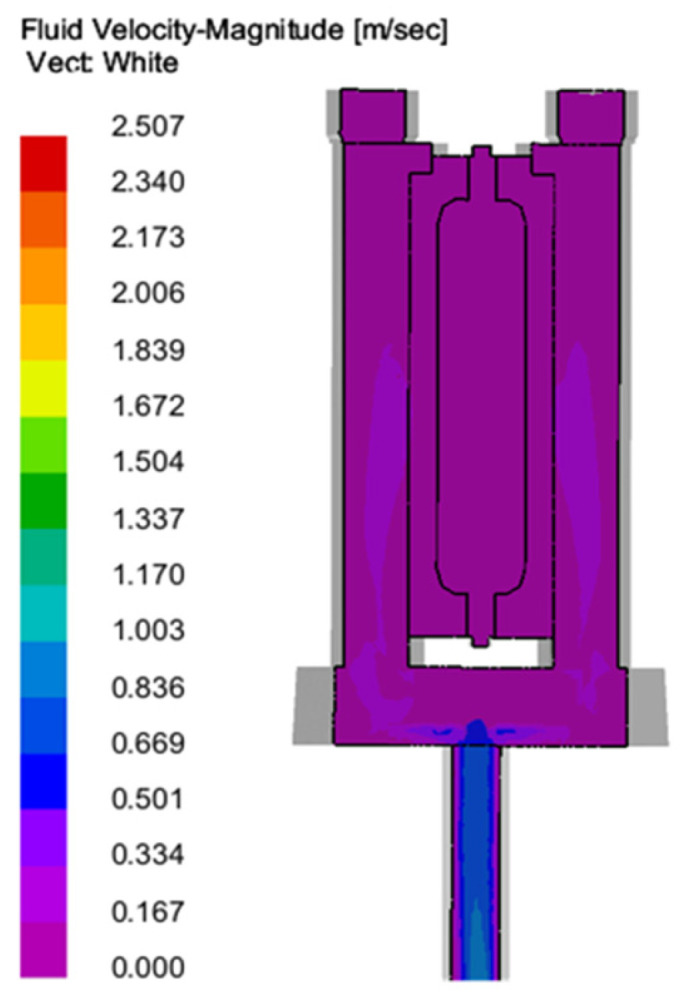
Flow velocity of metal liquid during casting (*T* = 30 s).

**Figure 9:**
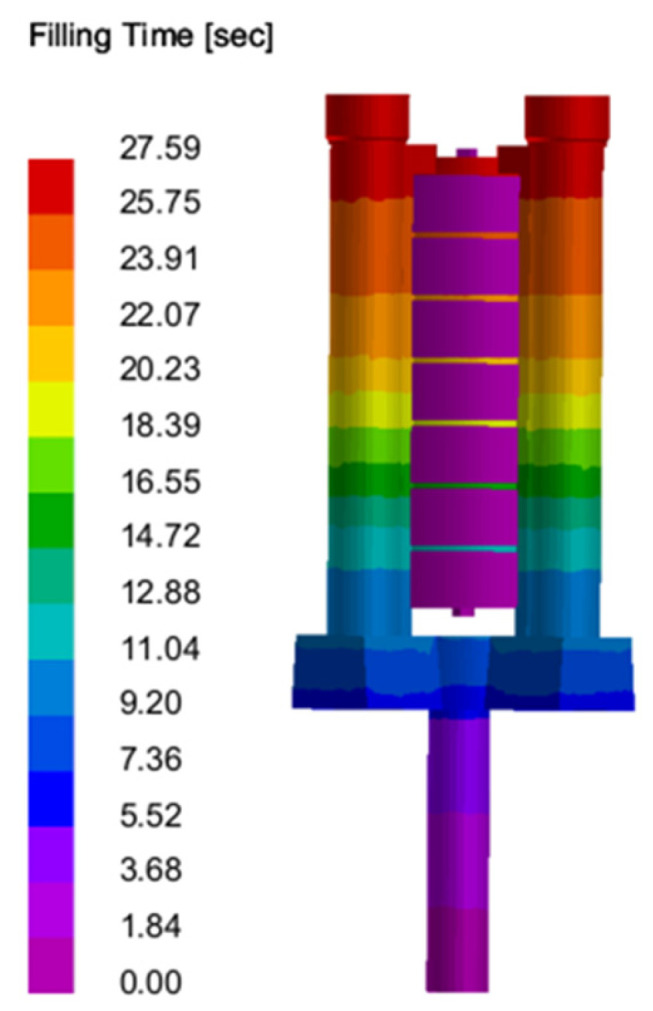
Filling time of metal liquid during casting.

Filling: The column gap pouring optimization system designed in this study is shown in Figure 6. Through the analysis of filling simulation results, the following can be seen (as shown in Figure 7, Figure 8 and Figure 9): with the passage of time, the impact force vector entering the cross runner changes smoothly and the filling speed is uniform in all parts (as shown in Figure 8), which is conducive to the gas exclusion in the casting cavity to avoid the defects such as liquid metal slag inclusion, porosity and gas wrapping. In addition, the filling layers in Figure 9 show different colors and are distributed horizontally in a band, and the filling is smooth and sequential solidification is realized.

**Figure 10:**
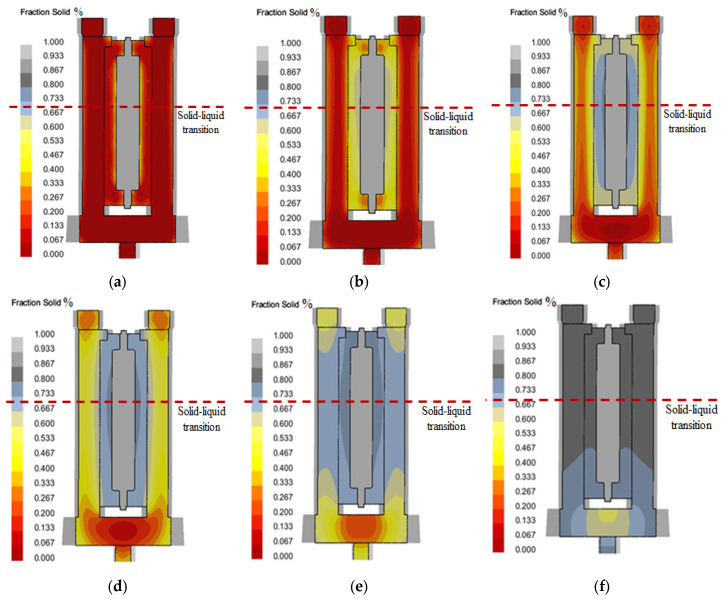
Simulation results of solid phase fraction during filling of column slot casting optimization system: (**a**) *T* = 200 s; (**b**) *T* = 400 s; (**c**) *T* = 800 s; (**d**) *T* = 1200 s; (**e**) *T* = 2000 s; (**f**) *T* = 3000 s.

Solidified: In combination with Figure 10, it can be seen that the addition of thermal insulation riser and cold iron ensures a better sequential solidification. At 1000 s, the whole casting begins to solidify in an inside–out sequence. In 1500 s, the casting is solidified first by the metal liquid at the gate of the column to supplement the casting; in 2500 s, the metal liquid of the column solidifies in the riser and the cross runner respectively. It ensures the effective feeding of the whole pouring system to the casting.

**Figure 11:**
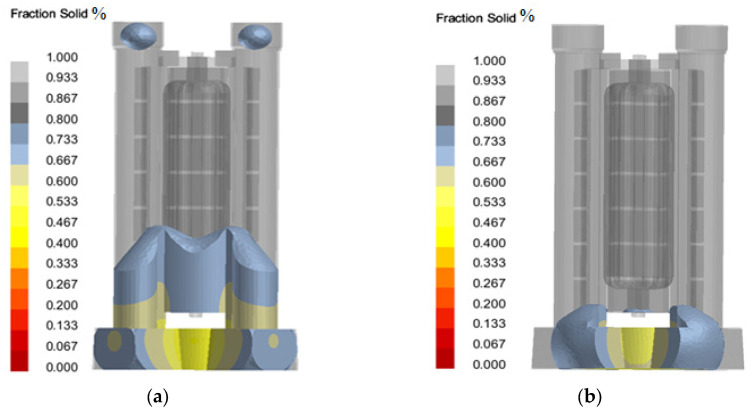
Overall heat node distribution during filling of the column slot casting optimization system: (**a**) top hot knot; (**b**) bottom hot knot.

In order to better verify the correctness of the solidification sequence, the location of the hot knot (that is, the maximum thickness at the intersection of two walls or multiple walls) was found for verification, the solid phase fraction was set to 0–0.7, only the solidified region was displayed, and then the final solidified region of the liquid metal was viewed. The overall position of the hot knot was shown in Figure 11. The hot knot is mainly concentrated in the riser and the cross runner, which is consistent with the simulation results shown by the solid phase fraction during solidification process. Only a few “isolated” melting zones are produced in the casting cavity, which can be eliminated by further optimizing the casting process parameters, which proves the rationality of the pouring system design.

**Figure 12:**
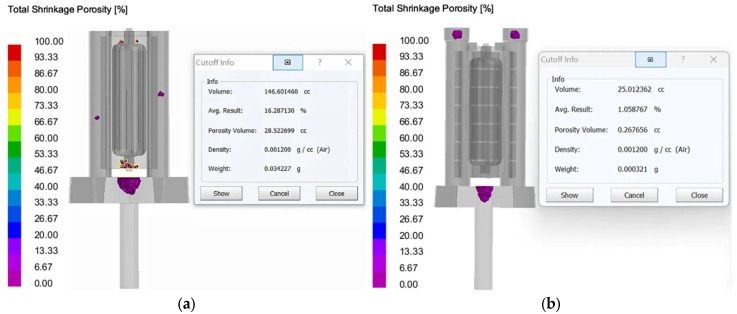
The distribution of shrinkage and porosity of the finished parts after forming with the column slot casting optimization system: (**a**) traditional design; (**b**) improved design.

Shrinkage and porosity: As shown in Figure 12, the optimized casting system has no shrinkage porosity and porosity defects at the bottom of the casting, and the total shrinkage porosity and porosity ratio inside the casting is 0.26 cc, which is 28.23 cc (maximum limit) lower than that of the traditional scheme. The reduction in defects is very obvious, which verifies the rationality of structural optimization. However, there are still a few defects in the low end of the casting, which need to be further optimized by selecting the optimal casting process parameters.

### 3.2. Optimization of Casting Process Parameters of Column Gap Casting Optimization System

In order to further improve the casting quality to achieve to eliminate the remaining defects, in addition to optimizing the structure of the casting system to improve the quality of the casting, the reasonable selection of the casting process parameters will also have a significant impact on the casting quality. Considering that shrinkage and porosity are the main defects in the casting process, the optimization of the casting process parameters studied in this paper takes shrinkage and porosity as the most important judgment basis. The method is as follows: firstly, a single-factor experiment is carried out to determine the mapping relationship between the casting process parameters and shrinkage and porosity, and the main factors leading to casting shrinkage and porosity are selected according to the single-factor experiment results and actual production experience, and the optimal range of each factor is determined. Secondly, the parameters are optimized by orthogonal experiment and response surface experiment. Finally, ProCast simulation was used to calculate the shrinkage and porosity of the castings and verify the reliability of the optimization.

#### 3.2.1. Single-Factor Experimental Design of Casting Process Parameters

Combined with a large number of early experimental studies on the influence of single-factor process parameters (which have a key impact on the casting quality in the casting process such as pouring temperature, liquid rise rate, dwell pressure, preheating temperature, pressure holding time) on the microstructure and comprehensive mechanical behavior of the cast state, this study established the following relationship between the influence of each process parameter on the porosity through numerical simulation analysis and joint verification test; at the same time, a more detailed mechanism of action is also included in our previous study [22].

**Figure 13:**
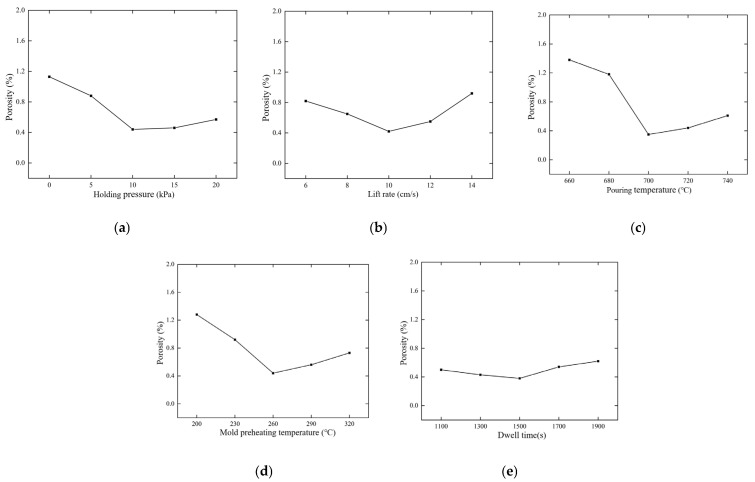
Influence of process parameters on porosity: (**a**) holding pressure; (**b**) liquid rise rate; (**c**) pouring temperature; (**d**) mold preheating temperature; (**e**) pressure-holding time.

(1) The porosity of castings under different holding pressures was simulated and predicted, and the results were shown in Figure 13a. With the increase in holding pressure, the porosity decreases first and then flattens. When the holding pressure is increased from 5 to 10 kPa, the porosity decreases obviously, this is because the casting can be effectively supplemented by the metal liquid under the pressure during the solidification process, which makes the crystal structure of the casting denser and the grain finer. However, after the increase of 15 kPa, the porosity of the metal liquid under the action of pressure decreases gently, indicating that the influence of the holding pressure on the porosity is no longer obvious after 10 kPa. In order to obtain a lower porosity, greater pressure is needed, but the processing cost is also increased. Therefore, 10 kPa is selected as the best pressure holding.

(2) The porosity of the casting was simulated and predicted at different liquid metal lifting speeds in the liquid lifting stage, and the results were shown in Figure 13b. The porosity of the casting decreases first and then increases with the increase in the liquid metal lifting rate, and the overall process is relatively smooth and the change is not significant. This is because when the liquid lift rate is too low, cold isolation is easy to occur during the filling process for too long, and oxidation and porosity are easy to occur when the contact time with the air is too long. When the casting porosity reaches 10 cm/s, the casting porosity is reduced to the minimum. When the lifting speed is too fast, the filling pattern of the metal liquid will be unstable, and the phenomenon of splashing and turbulence will occur, so the best lifting speed of the metal liquid is 10 cm/s.

(3) The porosity of castings at different pouring temperatures was simulated and predicted, and the results were shown in Figure 13c. The porosity of the casting decreases with the increase in the pouring temperature, and the porosity of the casting reaches the minimum when the pouring temperature of the metal liquid reaches about 700 °C. As the casting temperature continues to increase, the porosity of the casting increases gradually. This is because the temperature of the liquid metal is too high, the grain growth is coarser and the solidification time is longer, the larger thermal stress and thermal shock will be generated during the filling solidification process, and a large number of high temperature gases will be generated to form shrinkage holes. After comprehensive consideration, 700 °C was determined as the best pouring temperature.

(4) The porosity of castings under different mold preheating temperatures was simulated and predicted, and the results were shown in Figure 13d. The porosity of castings decreases rapidly at first and then increases slowly with the increase in mold preheating temperature. This is due to the fact that at a small preheating temperature, sufficient heat cannot be provided to maintain the fluidity of the metal liquid during the pouring process, increasing the risk of cold isolation. A proper increase in mold temperature can slow down the solidification cooling rate, which is conducive to the removal of gas. When the casting reaches 260 °C, the casting porosity reaches the lowest. As the mold temperature increases, it will cause the moisture and volatile substances present in the mold material to evaporate faster, producing more gas. These gases are trapped in the metal liquid during the casting process, resulting in the phenomenon of gas encapsulation, and the formation of more pores and shrinkage holes. Therefore, the optimal preheating temperature of the mold is 260 °C.

(5) The porosity of castings under different holding times was simulated and predicted, and the results were shown in Figure 13e. The porosity of the casting decreases slowly at first and then increases slowly with the increase in the holding time. This is because the appropriate pressure holding time can make the casting can be better supplemented, but the pressure-holding time is too long with the further increase in the internal temperature of the casting, the casting grain growth will be larger, increasing the porosity of the casting. Overall consideration, the pressure-holding time has only a weak influence on the overall process. When it reaches 1500 s, the casting porosity reaches the optimal level, so 1500 s is the best pressure-holding time.

#### 3.2.2. Orthogonal Experimental Design of Casting Process Parameters

The orthogonal test is mainly designed for a large number of process parameters and their value range. It is an experimental design method based on mathematical statistics. It selects representative data points to achieve the minimum number of tests and conducts tests for multiple factors to obtain an accurate estimate of each factor and its interaction. According to the above single-factor experimental results, the fixed liquid rising speed is 10 cm/s and the holding time is 1500 s (the factors that played little role in the above single-factor influence experiment were fixed). The three factors that have the greatest influence on the casting porosity, including holding pressure (A), pouring temperature (B), and preheating temperature (C), are screened. In addition, the holding pressure of 10 kPa, pouring temperature of 700 °C and mold preheating temperature of 260 °C were taken as the central point, the left and right ends of the central point were taken as the value range of the level (a broader range of factor orthogonality is also studied in this study, but the regions with significant effects are mainly described in this study), porosity was selected as the evaluation index, and a three-factor and three-level orthogonal test was designed. The results of numerical simulation and further range analysis were shown in Table 4 and Table 5.

According to the R value (reflects the degree of influence of various process parameters on porosity. Table 5), the influence of each factor on the casting porosity is as follows: pouring temperature > mold preheating temperature > holding pressure.

#### 3.2.3. Response Surface Experiment Design of Casting Process Parameters

Response surface optimization is a type of multiple regression analysis based on studying interactions and ANOVA to better understand and optimize the factors that affect the casting or specific response [23,24]. In the response surface optimization test, a mathematical model is established to describe the mapping relationship between process parameters (i.e., factors) and casting quality (i.e., response), and the best combination of factors is determined to achieve the best response. There are many types of design model optimization methods for response surface analysis. According to the actual test conditions and purposes, BB, the central combination test, is suitable for experiments with only three levels, with fewer test times and higher calculation accuracy. This study selects three main influencing factors according to the Box–Behnken experimental design principle. The lowest level of holding pressure, pouring temperature and preheating temperature [25] of casting mold were denoted as −1, the highest level as 1, and the middle level as 0. Response surface software D-E 13.0 was used to carry out three-factor and three-level design, and then the best casting process parameters were determined. D-E 13.0 data processing software was used to input the corresponding factor levels to make the response surface test design table, and each factor and level was analyzed by BB model optimization design method. The response surface experiment will study the interaction between factors. A total of 17 groups of response surface experiments were conducted, among which 5 groups were central point repeated experiments. The purpose of the repeated experiment is to estimate the experimental error and investigate the fitting of the central region through the porosity experimental results of the repeated experiment. According to the horizontal combination of the test table, the experimental data measured by ProCast simulation are shown in Table 6 below.

Based on the above data, quadratic polynomial regression model was used to fit and analyze the grouped data. The significance test results of the regression model coefficient, various factors and interactions between factors were shown in Table 7. The regression equation of the model was obtained by applying the least square method to the experimental grouping and results of the response surface in Table 7:

(1) $$Y_{Porosity}=0.42-0.14\times A-0.41\times B-0.16\times C-0.33\times AB+0.2\times AC+0.053\times BC\;+0.049\times A^{2}+0.44\times B^{2}+0.31\times C^{2}$$

In order to accurately and reasonably evaluate the coupling effect of each casting process parameter by response surface method, the indexes Pre R^2^ and Adj R^2^ were introduced to evaluate the fitting degree and prediction ability of the regression model. Among them, R^2^ is the main variable to judge the fit degree of the model, and its value ranges from 0 to 1, where 0 indicates that the model cannot explain the variability of the dependent variable, and 1 indicates that the model can fully explain the variability of the dependent variable. The closer R^2^ is to 1, the better the model fits. However, a potential problem in evaluating the accuracy of model fitting using Formula (2) is that the increase in independent variables, even if they are not directly related to y, can lead to an inflated R^2^. To counter this “false increase”, statisticians introduce an Adj R^2^ (shown in Formula (3)). The Adj R^2^ is an improvement on Pre R^2^, with both the numerator and denominator adjusted, where *p* represents the number of independent variables (which is an indicator used to judge the significance of F-value) and n is the total number of samples. This adjustment amounts to a “penalty” for a model with too many independent variables, ensuring that the increase in variables does not simply increase the R^2^ value, but rather pursues more precise explanatory power and model simplification. Meanwhile, according to reference [26,27], the model has excellent data fitting when Adj R^2^ ≥ 0.8, good fitting when 0.6 ≤ Adj R^2^ ≤ 0.8, medium fitting when 0.4 ≤ Adj R^2^ ≤ 0.6, and poor fitting when Adj R^2^ ≤ 0.4.

(2) $$\mathrm{Pre}{\;R}^{2}=\frac{SSR}{SST}$$

(3) $${\mathrm{Adj}\;R}^{2}=\frac{SSR/P}{SST/n-P-1}$$

In the formula, SST is the sum of the squares of the deviation between the observed value of the dependent variable and the mean, and SSR is the sum of the squares of the regression, which is the deviation caused by the variable x.

**Figure 14:**
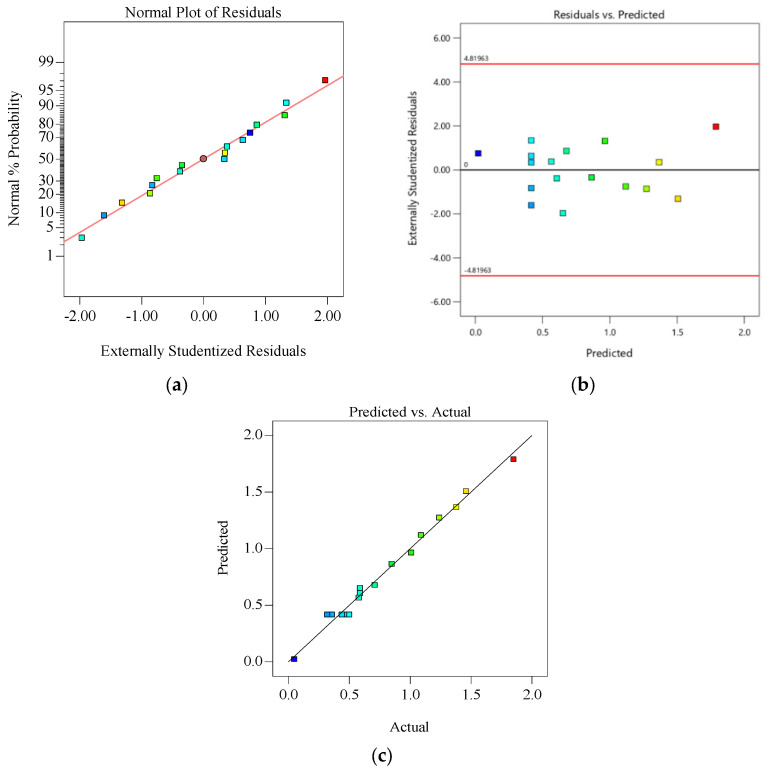
Model diagnosis diagram of casting defect prediction model: (**a**) residual distribution characteristics; (**b**) residuals prediction; (**c**) forecast and actual.

Combined with the results in Table 7, it can be seen that Adj R^2^ = 0.9761, Pre R^2^ = 0.9192, and the Adj R^2^ is very close to 1, indicating that the Formula (1) model established in this study has a high accuracy and can accurately predict the casting defects under the coupling of various casting process parameters. Meanwhile, if the *p*-value of the whole model is <0.0001, that is, *p* < 0.05, it indicates that this regression model extremely significantly meets the statistical law. Among them, the two secondary interaction terms AB and AC, whose *p* values are <0.0001 and 0.0012 (*p* < 0.05), have a very significant impact on casting defects; BC, whose *p* values are 0.1987 (*p* > 0.05), has no significant impact on casting defects. The interaction between casting process parameters has the most significant influence on casting defects (porosity) as holding pressure and pouring temperature AB of liquid metal (AB > AC > BC). In addition, the correlation image distribution of the model can be verified again: as shown in Figure 14a, residuals of the porosity parameter optimization model are located around the predicted line, indicating that the model meets normal distribution; residual prediction graph in Figure 14b are scattered and irregularly distributed on both sides of the line; all points are distributed on a straight line in both the predicted and actual figures of Figure 14c. Through analysis, the established porosity parameter optimization model meets the model diagnostic criteria, indicating that the model is reliable and meets the statistical law.

**Figure 15:**
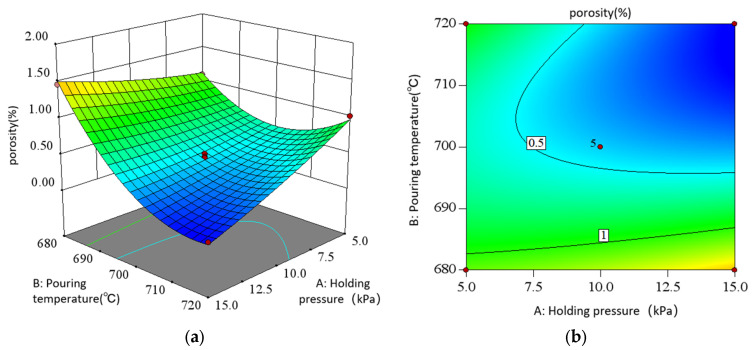
Interaction of AB in response to porosity: (**a**) surface diagram; (**b**) contour diagram.

As shown in the direct view of the interaction in Figure 15, when the holding pressure is low at 5 kPa, the porosity shows a trend of slowly decreasing at first and then increasing with the increase in pouring temperature. When the holding pressure is high, the porosity decreases first and then flattens with the increase in pouring temperature. When the pouring temperature is low, the porosity increases slowly with the increase in the holding pressure. When the casting temperature is high, the porosity decreases with the increase in holding pressure. It can be seen that there is a significant interaction between the holding pressure and the pouring temperature of the metal liquid in low pressure casting. In contrast, the longitudinal span and contour gradient of the response surface vary greatly in the direction of pouring temperature, which indicates that pouring temperature has a more significant effect on the porosity than holding pressure. When the holding pressure is 7.5–12.5 kPa and the pouring temperature is 700–720 °C, the porosity can be significantly reduced.

**Figure 16:**
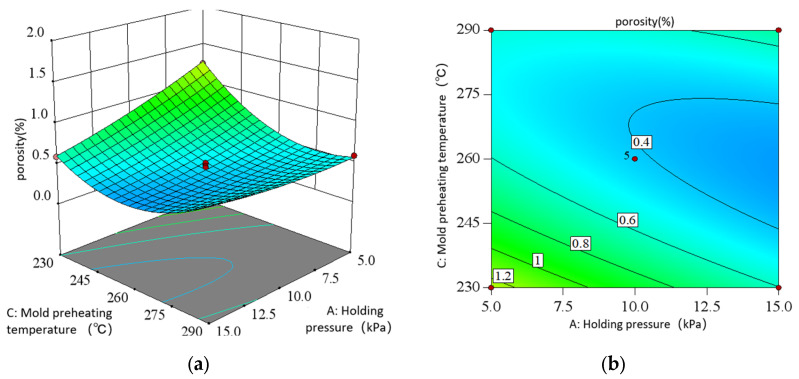
Interaction of AC in response to porosity: (**a**) surface diagram; (**b**) contour diagram.

As shown in the direct view of the interaction in Figure 16, with the increase in the holding pressure, the porosity decreases first and then decreases almost gently; with the increase in the preheating temperature of the mold, the porosity decreases first and then increases. Moreover, when the holding pressure is different, the porosity change amplitude is different with the preheating temperature of the mold. This indicates that there is a significant interaction between the holding pressure and the preheating temperature of the mold. In contrast, the directional surface fluctuation of the preheating temperature of the mold is larger, indicating that its influence on the porosity is more significant than that of the holding pressure. Only considering the interaction of the two, the optimal porosity process conditions are concentrated in the holding pressure of 7.5–12.5 kPa and the preheating temperature of the mold is 245–275 °C.

**Figure 17:**
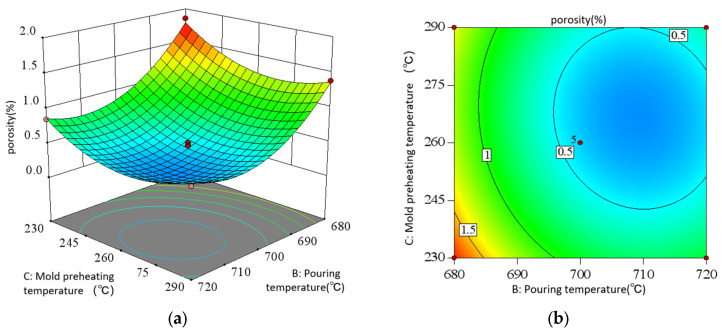
Interaction of BC in response to porosity: (**a**) surface diagram; (**b**) contour diagram.

As shown in the direct view of the interaction in Figure 17, when the holding pressure is 10 kpa (0 level), the porosity of the casting tends to decrease slowly at first and then increase as the temperature of molten metal pouring rises, and decreases first and then increases as the preheating temperature of the casting increases. Considering only the interaction of the two, the optimum critical process parameters of porosity are when the pouring temperature is around 700–720 °C and the preheating temperature of the casting mold is around 245–275 °C.

Finally, the optimization module of the model was selected by D-E 13.0 to set the optimal target of the model in the numerical value, and the minimum value was selected for optimization calculation. According to the operation results of the D-E 13.0 software, the optimal process of casting defects under the common influence of various factors and levels is as follows: the holding pressure is 14.68 kPa, the pouring temperature is 717.152 °C, and the preheating temperature of the casting mold is 256.12 °C. Under these conditions, the porosity predicted by the model is 0.006%, which is almost zero to achieve the optimal value.

#### 3.2.4. Verification of Optimal Parameters of Casting Process

**Figure 18:**
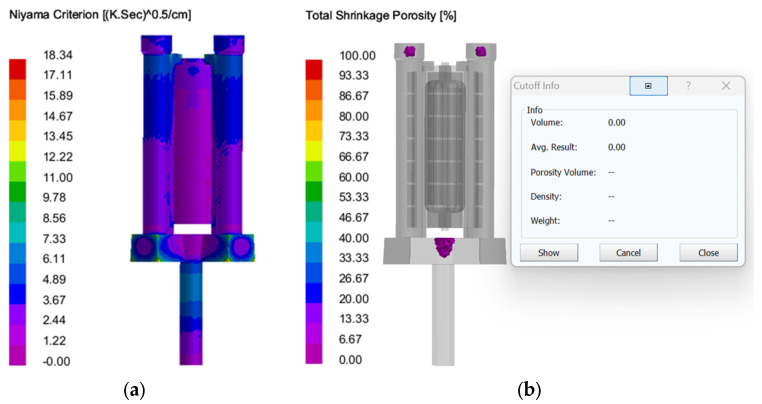
Casting defects under the optimal combination of process parameters: (**a**) results of Niyama Criterion; (**b**) results of porosity.

**Verification of shrinkage and porosity:** The Procast casting simulation and prediction was carried out through the optimized process parameter combination, as shown in Figure 18, indicating that the overall casting would not appear significantly loose, and the defect ratio was reduced by 1.05% compared with that before the process parameter optimization, indicating a significant reduction in casting defects, which verified the rationality of response surface analysis to optimize the optimal process parameters. Meanwhile, in order to facilitate reading, the simulation results of casting defects under the above orthogonal test and optimization parameters are summarized in Table 8.

**Figure 19:**
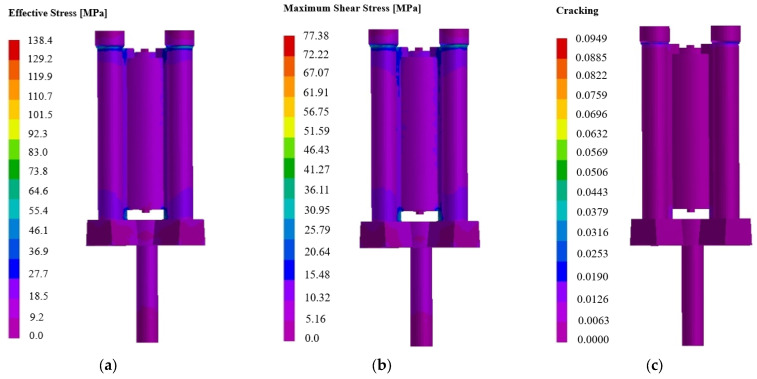
Simulation results of stress during casting solidification under optimal process parameters: (**a**) effective stress; (**b**) maximum shear stress; (**c**) crack analysis.

**Figure 20:**
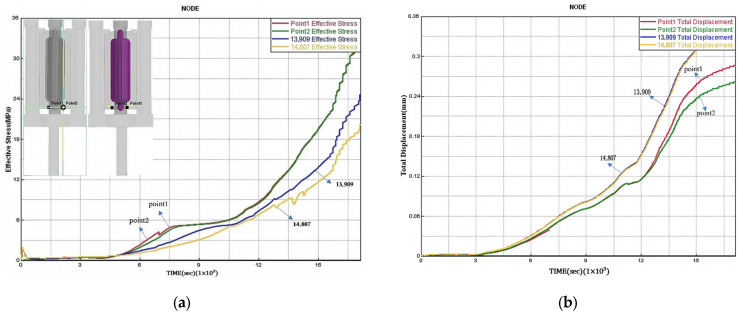
Simulation results of node stress during casting solidification under optimal process parameters: (**a**) effective stress distribution; (**b**) node strain distribution.

**Figure 21:**
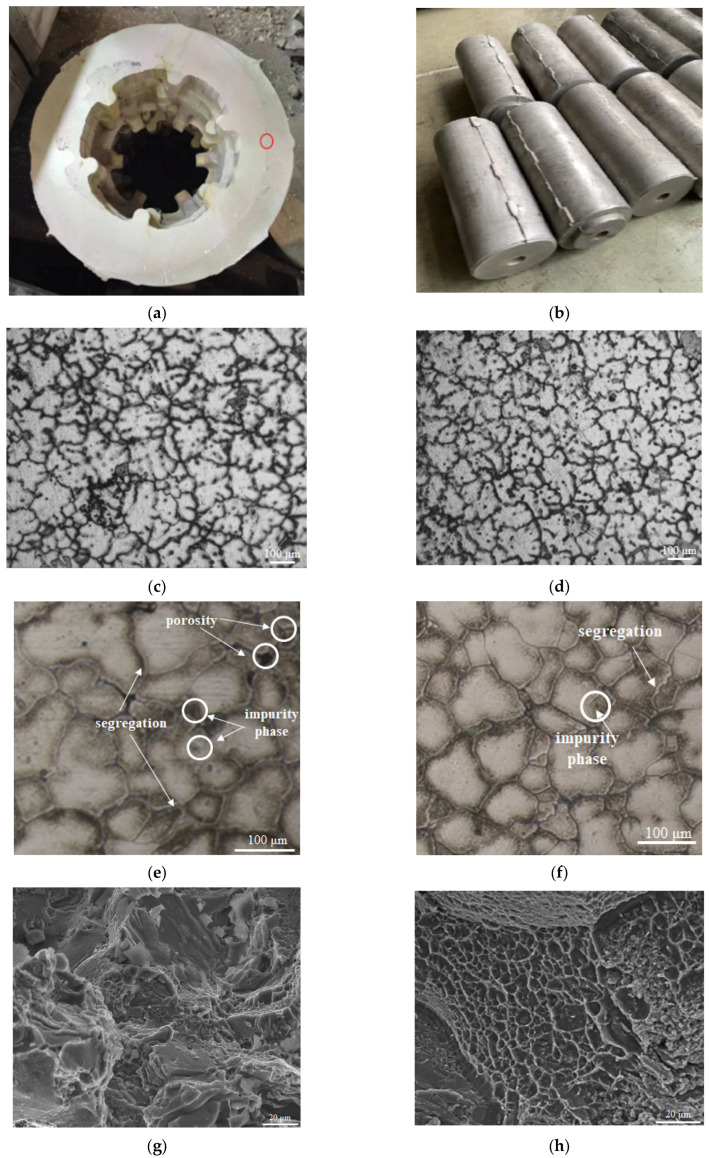
Comparison of microstructure of two casting methods: (**a**) casting body (top surface) with optimized process parameters; (**b**) casting body (side surface) with optimized process parameters; (**c**) low magnification of crystal structure (original process scheme); (**d**) low magnification of crystal structure (optimized process scheme); (**e**) high magnification of phase (original process scheme); (**f**) high magnification of phase (optimized process scheme); (**g**) tensile fracture morphology (original process scheme); (**h**) tensile fracture morphology (optimized process scheme).

**Verification of thermal stress defect:** Based on the optimal process parameters obtained above, the ProCast stress module is used to conduct corresponding coupling simulation of temperature field and stress field, mainly analyzing the distribution of internal stress, deformation and crack during the casting process, and verify whether the casting deformation to meet the product requirements of cylinder castings. The post-processing analysis results are shown in Figure 19, Figure 20 and Figure 21.

Observe the distribution of the effective stress of the casting in Figure 19a, the effective stress value is 9.2–27.7 MPa, and the stress is within the tensile strength range of ZL205A alloy castings. See Figure 19b, the maximum shear stress generated inside the casting during the casting process is 5.16–15.48 MPa, and the formation of a crack has a greater impact on the hot crack. Due to the low tensile strength of the alloy at high temperature, if the linear shrinkage is hindered during the shrinkage and solidification of the casting and the stress exceeds the strength of the alloy, the hot crack phenomenon will occur. According to the crack analysis in Figure 19c, it can be seen that the casting will not produce cracks and other defects, which meets the requirements of product casting. According to the structural characteristics of the castings studied in this paper, the casting internal stress is more likely to occur where the wall thickness is uneven and the thick hot zone is large. The cross section between the bottom of the casting and the sand core is the key part of the overall service of the casting, which is more prone to stress concentration problems, and the stress distribution at the bottom of the casting should be emphasized. Finally, by randomly establishing four nodes at the bottom of the casting, the distribution relationship of the effective stress and strain at the internal nodes of the casting is further summarized as follows: combined with the stress–strain results shown in Figure 20, it can be seen that the internal and external stress and strain of the key service parts of the research object (that is, the cross section between the bottom of the casting and the sand core) have a high consistency, and the range of stress and strain is 0–31 MPa and 0–3 mm, respectively, so the stress at the key position at the bottom of the casting is within the tensile strength range of ZL205A alloy. For the deformation of the casting, the strain belongs to the microscopic deformation, and the set machining allowance is enough to cover the deformation of the casting.

**Verification of microstructure properties and fracture toughness:** In order to further verify the quality improvement in the optimized process parameters proposed by the response surface method in the casting of large long lead cylinder of aluminum alloy, the following comparisons were made with the forming microstructure phase distribution and fracture toughness under the traditional unoptimized process parameters: based on the optimal combination of process parameters obtained in this study, the large long lead cylinder of aluminum alloy cast body shown in Figure 21a,b was produced by casting. By wire cutting the samples of the non-optimized process and the optimized process, Leica optical microscope (model: DM2500P + 7HMS, Leica Microsystems Wetzlar GmbH, Wetzlar, Germany), scanning electron microscope (model: Philip-Quanta 400 F, PHILIPS/FEI Commpany, Hillsboro, OR, USA) and tensile tester (model: SC-J-500, Mitutoyo Ltd., Kawasaki, Japan) were successively used to conduct comparative analysis of the crystal structure, phase and fracture morphology of the formed microstructure, as shown in Figure 21c–h. It can be seen from Figure 21c,d that the optimized process parameters obtained a smaller overall size of cellular crystals through the optimal coordination of each pouring parameter, which combined with the grain boundary dislocation movement mechanism would have higher mechanical properties. In Figure 21e,f with further magnification, it can be seen that, on the one hand, the microstructure without optimized parameters has intergranular porosity, which will reduce the comprehensive mechanical properties of the cast. On the other hand, due to the unreasonable selection of process parameters, more impurity phases (mainly composed of Al, Cu and Ti) and segregation are formed because of insufficient solid solution of intergranular trace elements into the crystal. The optimized structure has better mechanical properties than the unoptimized structure because more intergranular impurity phase is dissolved in solid solution. Meanwhile, it can be seen from the fracture morphology in Figure 21g,h, the fracture morphology of the material with optimized parameters presents a more deep and smaller dimple than that of the material without optimized parameters, which further verifies the effect of the above microscopic structures on the macroscopic mechanical behavior. In addition, this paper also compares the optimal results achieved by this technology with the two existing traditional casting processes (as shown in Table 9), thus verifying the accuracy of this research method.

## 4. Conclusions

As the core component of chain-less ammunition transmission system, large long lead cylinder adopts ZL205A alloy, which has the advantages of high strength and wear resistance. In order to achieve high-quality manufacturing for the columns of this structure, this paper based on the optimized gap pouring system of the previous design, the reasonable range of casting process parameters was determined by single-factor analysis. Secondly, by setting the orthogonal test in this range and conducting the numerical simulation of casting defects, the simulation results of casting defects under the combination of various process parameters are obtained. Furthermore, by introducing the response surface method, a quantitative analysis model (Adj R^2^ = 0.9761) was established to map the casting defects of each process parameter combination in a quantitative form, and the optimal process parameters were obtained as follows: holding pressure 14.68 kPa, casting temperature 717.152 °C and mold preheating temperature 256.12 °C.

Finally, by comparing with the casting entity without optimized process parameters, the following is found:

(1) Compared with the non-optimized cast material, the optimized cast material exhibits higher fracture toughness due to its finer crystal structure, fewer intergranular pores and intergranular segregation.
(2) Compared with traditional metal mold casting and unoptimized low-pressure casting, the tensile strength of non-porous casting with pressure-holding pressure 14.68 kPa, casting temperature 717.152 °C and mold preheating temperature 256.12 °C increased by 6.6% and 4.1%, respectively, hardness increased by 14.3% and 8.4%, respectively, and the elongation is increased by 16.9% and 10.6%, respectively, thus efficiently and accurately improving the process quality.

Based on the above technology, high efficiency and high-quality casting is realized. The technical method proposed in this study is mainly applicable to the process parameter optimization in the field of industrial manufacturing (that is, multiple process factors map a single index). In addition, due to the use of numerical simulation analysis technology to predict casting defects, the research and development cycle and cost of the process are greatly reduced, and accurate quantitative design is achieved. However, since the optimization model of process parameters obtained by the response surface method is still based on more experimental results for fitting calculation, how to build a better algorithm model and achieve more accurate and efficient feedback design with less data, and multi-factor optimization multi-objective will be an important research direction in the future.

## Figures and Tables

**Table 1:** Pressure and speed at each stage of low-pressure casting process.

	Lifting Liquid	Mold Filling	Turbocharge	Pressure Maintaining	Pressure Relief
Time t (s)	7	13.3	4	1500	1
Pressure *P* (kPa)	13.82	29.82	39.82	39.82	0
Rate of pressure increasing *V* (kPa/s)	1.5	1.2	2.5		
Rising velocity *V* (cm/s)	10	6			

**Table 2:** Heat transfer coefficient.

Heat Exchange Interface Type	Mold—Sand Core	Alloy—Cold Iron	Riser Pipe—Cast	Cold Iron—Cast	Riser—Cast
Coefficient of heat transfer H [W/(m^2^·K)]	H = 500	H = 2000	H = 20	H = 500	H = 20

**Table 3:** Quality comparison under different mesh partitioning methods.

		Meshing Method	Mesh Minimum Size	Mesh Maximum Size	Partition Quantity	Minimum Jacobian	Computation Time
Plan 1	Surface Mesh	Triangular Mesh	0.6 mm	1 mm	58,006	0.62	<1 h
	Volume Mesh	Hexahedral Mesh	0.6 mm	1 mm	905,338	0.65	
Plan 2	Surface Mesh	Triangular Mesh	0.6 mm	1 mm	85,884	0.77	1.5 h
	Volume Mesh	Hexahedral Mesh	0.6 mm	1 mm	1,604,376	0.78	
Plan 3	Surface Mesh	Triangular Mesh	0.6 mm	1 mm	198,276	0.94	2 h
	Volume Mesh	Hexahedral Mesh	0.6 mm	1 mm	3,259,789	0.92	

**Table 4:** Orthogonal test table of casting process parameters.

Experiment Number	A Pressure-Holding Pressure/kPa	B Pouring Temperature/°C	C Mold Preheating Temperature/°C	Porosity/%
1	5	680	230	1.16
2	5	700	260	0.88
3	5	720	290	1.21
4	10	680	290	1.38
5	10	700	230	0.92
6	10	720	260	0.45
7	15	680	260	1.46
8	15	700	290	0.71
9	15	720	230	0.52

**Table 5:** Range analysis of influence of casting process parameters on porosity.

Range R	A Pressure-Holding Pressure/kPa	B Pouring Temperature/°C	C Mold Preheating Temperature/°C
K_1_	1.083	1.330	0.867
K_2_	0.917	0.837	0.930
K_3_	0.896	0.727	1.100
R	0.187	0.603	0.233

**Table 6:** Experimental grouping and results of response surface.

Test Number	A Pressure-Holding Pressure/kPa	B Pouring Temperature/°C	C Mold Preheating Temperature/°C	Porosity/%
1	0	0	0	0.46
2	−1	−1	0	1.09
3	1	1	0	0.05
4	0	0	0	0.32
5	−1	0	−1	1.24
6	0	−1	1	1.38
7	0	0	0	0.44
8	1	0	1	0.71
9	0	0	0	0.5
10	0	1	1	0.59
11	0	1	−1	0.85
12	−1	1	0	1.01
13	1	0	−1	0.59
14	0	0	0	0.36
15	−1	0	1	0.58
16	0	−1	−1	1.85
17	1	−1	0	1.46

**Table 7:** Results of variance analysis and regression of porosity model.

Source	Sum of Squares of Deviations	Degree of Freedom	Mean Square	F Value	*p* Value	Significance
Model	3.63	9	0.4	73.65	<0.0001	**
A—Holding pressure	0.15	1	0.15	28.15	0.0011	**
B—Pouring temperature	1.34	1	1.34	245.82	<0.0001	**
C—Mold preheating temperature	0.2	1	0.2	36.85	0.0005	**
AB	0.44	1	0.44	80.83	<0.0001	**
AC	0.15	1	0.15	27.8	0.0012	**
BC	0.011	1	0.011	2.02	0.1987	
A^2^	0.01	1	0.01	1.89	0.212	
B^2^	0.8	1	0.8	146.98	<0.0001	**
C^2^	0.42	1	0.42	76.13	<0.0001	**
Residue	0.038	7	5.478 × 10^−3^			
Missing fit	0.016	3	5.468 × 10^−3^	1	0.4803	ns
Pure error	0.022	4	5.488 × 10^−3^			
Sum	3.66	16				

Note: R^2^ = 0.9895, Adj R^2^ = 0.9761, Pre R^2^ = 0.9192, *p* < 0.01 is extremely significant, indicated by **, *p* < 0.05 is significant, indicated by *, *p* > 0.05 is not significant, indicated by ns.

**Table 8:** The distribution of casting defects under the combination of various process parameters.

Test Number	A Pressure-Holding Pressure/kPa	B Pouring Temperature/°C	C Mold Preheating Temperature/°C	Porosity/%
1	5	680	230	1.16
2	5	680	260	1.09
3	5	700	230	1.24
4	5	700	260	0.88
5	5	700	290	0.58
6	5	720	260	1.01
7	5	720	290	1.21
8	10	680	230	1.85
9	10	680	290	1.38
10	10	700	230	0.92
11	10	700	260	0.46
12	10	700	260	0.32
13	10	700	260	0.44
14	10	700	260	0.5
15	10	720	230	0.85
16	10	720	260	0.45
17	10	720	290	0.59
18	15	680	260	1.46
19	15	700	290	0.71
20	15	700	290	0.71
21	15	720	230	0.52
22	15	720	260	0.05
23	14.68	717.152	256.12	0.006 Best

**Table 9:** Comparison of the characteristics of each casting process.

Casting Method	Structure Design of Casting System	Porosity/%	Tensile Strength/MPa	Elongation /%	Hardness/HV
Traditional metal casting	Two serpentine straight runner, inner runner and cross runner riser	1.2%	472	7.1	147
Traditional low-pressure casting	Single side column straight runner, cross runner and ladder inner runner	0.65%	483	7.5	155
New low-pressure casting	Thermal insulation riser, slot type inner runner, column type straight runner and cold iron	0%	503	8.3	168

## Data Availability

The original contributions presented in the study are included in the article, further inquiries can be directed to the corresponding author.
